# Acupuncture Relieves the Excessive Excitation of Hypothalamic-Pituitary-Adrenal Cortex Axis Function and Correlates with the Regulatory Mechanism of GR, CRH, and ACTHR

**DOI:** 10.1155/2014/495379

**Published:** 2014-03-11

**Authors:** Shao-Jun Wang, Jiao-Jiao Zhang, Li-Li Qie

**Affiliations:** Institute of Acupuncture and Moxibustion, China Academy of Chinese Medical Sciences, Beijing 100700, China

## Abstract

It had been indicated in the previous studies that acupuncture relieved the excessive excitation of hypothalamic-pituitary-adrenal cortex axis (HPAA) function induced by stress stimulation. But the changes in glucocorticoid receptor (GR) induced by acupuncture have not been detected clearly. The objective of the study was to observe the impacts of acupuncture on the protein expressions of corticotrophin releasing hormone (CRH), adrenocorticotropic hormone receptor (ACTHR), and GR under the physiological and stress states. The results showed that under the stress state, acupuncture upregulated the protein expression of GR in the hippocampus, hypothalamic paraventricular nucleus (PVN), and pituitary gland, downregulated the protein expression of GR in the adrenal cortex, and obviously reduced the protein expressions of CRH and ACTHR. Under the physiological state, acupuncture promoted GR protein expression in the hippocampus and CRH protein expression in the hippocampus and PVN. The results explained that acupuncture regulated the stress reaction via promoting the combination of glucocorticoids (GC) with GR, and GR protein expression. The increase of GR protein expression induced feedback inhibition on the overexpression of CRH and ACTHR, likely decreased GC level, and caused the reduction of GR protein expression in the adrenal cortex.

## 1. Introduction

The normal expression of glucocorticoid receptor (GR) plays an important role in maintaining the normal physiological condition in the body [1]. A large number of studies had shown that the stresses induced by various reasons may cause the decrease of GR expression in the human body, rat, and dog, which is relevant to the stress intensity [2–4].

When the stressors arrive to the paraventricular nucleus of the hypothalamic paraventricular nucleus (PVN), corticotrophin releasing hormone (CRH) neurons synthesize and release CRH and transport it to the adenohypophysis. Afterward, CRH enables the adenohypophysis to secrete adrenocorticotropic hormone (ACTH). In blood circulation, ACTH acts on the adrenal cortex and accelerates the synthesis and the secretion of GC in the adrenal cortex. Being the negative feedback regulator, the ectogenic GC adjusts CRH secretion in the PVN through hypothalamic-pituitary-adrenal cortex axis (HPAA), inhibits the stress reaction, modifies the structure of the hippocampus and other limbic systems, and impacts awakening, cognitive, and emotional functions [5, 6]. The negative feedback regulation of GC on HPAA is achieved by the action on GR and mineralocorticoid receptor (MR) extensively distributed in the central nervous system [7].

Acupuncture is a procedure in which fine needles are inserted into an individual at discrete points and then manipulated, with the intent of regulating HPAA function [8–10]. The clinical practice of acupuncture is growing in popularity worldwide. More than 40 disorders have been endorsed by the World Health Organization (WHO) as conditions that can benefit from acupuncture treatment [11–13]. Acupuncture at Shenshu (BL23), Qimen (LR14), Sanyinjiao (SP6), Zhubin (KI9), or the other points regulates HPAA function, but the effects of different acupoints are different [14–17].

In this study, we discussed the impacts of acupuncture at different acupoints on GR expressions in the hippocampus, PVN, pituitary gland, and adrenal cortex of the rats induced by unpredictable chronic mild stress (UCMS) stimulation as well as the associated changes in the expressions of CRH and ACTHR so as to explore the acupoint specificity and the mechanism of acupuncture on the regulation of HPAA function.

## 2. Materials and Methods

### 2.1. Animals

Male Sprague-Dawley rats in 150–170 g were obtained from the Laboratory Animal Resources Center, National Institute for the Control of Pharmaceutical and Biological Products, Beijing (Certificate no. SCXK (jing) 2009–0017). These animals were individually caged on a 12 h light/dark cycle (lights on at 8:00 am, lights off at 8:00 pm) under controlled temperature (22 ± 1°C) and humidity (50% ± 5%) conditions. Standard rat chow and water were given ad libitum. Animals were allowed to acclimatize for seven days before the study. All experiment procedures comply with the guidelines of the “Principles of Laboratory Animal Care” (NIH publication no. 80-23, revised 1996) and the legislation of the People's Republic of China for the use and care of laboratory animals. The experimental protocols were approved by the Animal Experimentation Ethics Committee of the Institute of Acupuncture and Moxibustion, China Academy of Chinese Medical Sciences. Efforts were made to minimize the number of animals used and the suffering of the experimental animals.

### 2.2. Behavior Test

*Open Field Test.* The open field apparatus was constructed of black plywood and measured 80 × 80 cm with 40 cm walls. White lines were drawn on the floor. The lines divided the floor into twenty-five 16 × 16 cm squares. A central square (16 cm × 16 cm) was drawn in the middle of the open field. Rats were put on the central square; at the same time the video camera was turned on for video recording from the top of the open field apparatus. Behaviors of rats were recorded for 3 minutes, with the grid number being counted as the horizontal score and the time of both frontal claws uplifting from the ground as the vertical score, one score counted after every 10 cm moving along the specific lines [18, 19]. Only one animal was used in one determination. Each determination lasted 3 min. The next determination with another rat began after thoroughly cleaning the box. Each animal was determined before and after modeling, as well as after acupuncture separately.

*Sucrose Consumption.* The 24-hour water fasting was required in all the rats before and after modeling, as well as after acupuncture (after open field test), but 1% sucrose water was allowed freely for 24 h. The sucrose consumption was observed in 24 h for the animals of each group.

### 2.3. Establishment of Unpredictable Chronic Mild Stress (UCMS) Model Rat

Fifty-eight of one hundred rats were recruited with the total score of 60~120 in the open field test [18] and evenly randomized into 5 groups. A successful UCMS model rat was created with the score of the open field test equal to or minus 60. Qualified rats were distributed into 5 groups: a normal group (N group, no acupuncture, *n* = 10), a Qimen (LR14) and Shenshu (BL23) group (NEA group, *n* = 12), an UCMS group (M group, no acupuncture, *n* = 12), an UCMS Sanyinjiao (SP6) and Zhubin (KI9) group (MNEA group, *n* = 12), and an UCMS Qimen (LR14) and Shenshu (BL23) group (MEA group, *n* = 12). Every five rats in the N and NEA group were housed in one cage. However, rats in the M, MEA, and MNEA groups were caged individually. Depression model was established by 21 days of UCMS combined with isolation. UCMS procedures were based on published studies [19, 20], including 7 kinds of stressors: food deprivation, water deprivation, cage tilt 45°C (Ugo Basile s.r.l. hot/cold plate, Model 35100-001, Italy), swimming in 4°C ice water, clipping tail 3 min, 50 V electric shock (Electronic stimulator, NIHON KOHDEN, Japan), and overnight illumination. The stressors were given randomly 3 times daily for 21 continuous days. The rats in the N and NEA group were housed without any external stimuli except for necessary procedures such as routine cage cleaning.

### 2.4. Experimental Procedures (Figure 1)

**Figure 1:**
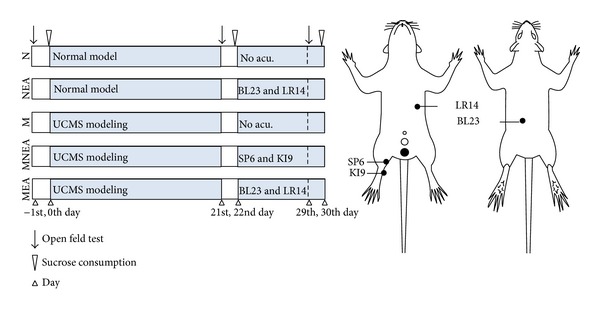
Experimental protocol. There were 5 groups. N: the normal group, no acupuncture; NEA: the normal acupuncture group, acupuncture at Qimen (LR14) and Shenshu (BL23); M: UCMS group, no acupuncture; MNEA: the group of UCMS acupuncture at Sanyinjiao (SP6) and Zhubin (KI9); MEA: the group of UCMS acupuncture at Qimen (LR14) and Shenshu (BL23) group.

The open field test on all rats was conducted on the day before the study (−1st day) , the 21st day (after UCMS), and the 29th day (after treatment) in the study course. After the models of UCMS were established on the 21st day, the electroacupuncture (EA) treatment of 8 days was applied to bilateral SP6 and KI9 of rats in the MNEA group once daily for 30 min. For the MEA group, EA was applied to bilateral BL23 and LR14 following the same procedure and EA parameters as the MNEA group. For the NEA group, EA was applied to the same procedure as the MEA group. M group and N group in the EA groups with the inhaled anesthesia accepted during the treatment (Figure 1). EA was applied at the frequency of 2 Hz and the intensity of 2 mA by using the EA stimulator (HANS-100A, Nanjing Gensun Medical Technology Co., Ltd., China). The inhaled anesthesia was conducted on the Isoflurane Vaporizer (Matrx VIP 3000, Midmark corporation, USA) with isoflurane (Hebei Nine Sent Pharmaceutical Co., Ltd., Hebei, China). Rats were sacrificed immediately after the last EA (on the 30th day), and the fresh tissues of brain (hippocampus and PVN area), pituitary, and adrenal gland were collected. Acupuncture, inhalation anesthesia process, or surgical procedure was the same in the NEA and the N group (Figure 1).

### 2.5. Western Blot Analysis

Western blot analysis was performed as follows [21]. Rats tissues of brain (hippocampus and PVN area), pituitary, and adrenal gland were homogenized on ice in RIPA buffer (50 mol/L Tris-Cl, pH 7.6, 5 mol/L ethylenediaminetetraacetic acid, 150 mol/L NaCl, 0.5% Nonidet P-40, and 0.5% Triton X-100) containing protease inhibitor cocktail and phosphatase inhibitor cocktails I/II (Sigma-Aldrich). The homogenate was centrifuged at 12,000 ×g for 30 minutes at 4°C. The supernatant was collected and the protein concentration was measured using the Bradford assay. Twenty micrograms of the sample was separated with 10% polyacrylamide gel blotted on a PVDF film (Millipore Corp.). The blotted film was blocked for 2 hours at 4°C in blocking solution (1×TBS with 5% nonfat milk and 0.02% Tween 20). The blocked film was shaken overnight at 4°C using primary antibodies in blocking solution. Following three times washes with TBST (1×TBS with 0.02% Tween 20), the film was shaken for 1 hour at room temperature with peroxidase-conjugated secondary antibody and then washed three times with TBST. Detection was performed using an ECL kit (Santa Cruz Biotech) according to the manufacturer's instructions. The western blots shown are representative of at least three independent experiments.

The antibodies used included the following: anti-CRH (Datashect, #10944-1-AP) (1 : 500), anti-ACTHR and anti-GR (Santa Cruz, #H-70 and #H-300) (1 : 1000), anti-*β*-Actin (Sigma, A5316) (1 : 10000), and HRP-conjugated IgG secondary antibodies (1 : 2000) (GE Healthcare Life Sciences). All western blot data were analyzed by ImageJ software.

### 2.6. Statistical Analysis

The statistical analysis was performed by using one-way analysis of variance (ANOVA) with software GraphPad Prism (California), and the data were expressed as means ± SEM. All results with *P* values less than 0.05 were considered statistically significant.

## 3. Results

### 3.1. Effects of Acupuncture on Behaviors in UCMS Model Rats

#### 3.1.1. Open Field Test (Figures 2(a), 2(b), and 2(c))

The activity of the animals in the five groups was gradually decreased on the 21st day (after modeling) and on the 29th day (after treatment) as compared with that before modeling (on −1st day). After modeling, the horizontal, vertical, and the along-the-line activities in the open field test in M, MNEA, and MEA groups were all reduced obviously as compared with N and NEA groups (*P* < 0.05, 0.01, and 0.001). After acupuncture, the difference in the horizontal activity was the most obvious as compared with that before acupuncture among the five groups. The decrease of the horizontal activity in M group was the most obvious, followed by MNEA, MEA, N, and NEA groups successively. The difference was extremely significant in M and MNEA groups as compared with N and NEA groups (*P* < 0.01). The difference was significant in MEA group as compared with N and NEA groups (*P* < 0.05) and between MEA and M groups (*P* < 0.05). The difference in the vertical activity was not significant among the groups. Concerning the along-the-line activity, the differences in M, MNEA, and MEA groups were significant extremely as compared with N and NEA groups (*P* < 0.001). There was no improvement after acupuncture.

#### 3.1.2. Sucrose Consumption (Figure 2(d))

The sucrose consumption was done on the 0th day (before modeling), on the 22nd day (after modeling), and on the 30th day (after acupuncture). After modeling, the results in M, MNEA, and MEA groups were different significantly as compared with N and NEA groups. After acupuncture, it was significantly reduced in M group, indicating the extremely significant differences as compared with N and NEA groups (*P* < 0.001). The difference was significant in MNEA group as compared with N and NEA groups (*P* < 0.05). The difference was not significant in MEA group as compared with N and NEA groups. The difference was extremely significant between MEA and M groups (*P* < 0.001). The difference was significant between MNEA and M group (*P* < 0.05) as well as between MEA and MNEA group.

There are results that showed that the stress reduced sucrose consumption (a symptom of anhedonia and considered a depressive-like behavior in rats) and 8 days of acupuncture, especially the MEA treatment, reduced anhedonia and restored the sucrose consumption of M animals to the levels of control animals. This is a very significant behavioral finding and should be discussed and properly emphasized.

### 3.2. Effect of Acupuncture on the Protein Expression of GR Induced by UCMS Stimulation (Figure 3)

Western blot analysis showed that there was a protein expression of GR detected in the hippocampus, PVN, pituitary gland, and adrenal cortex. GR protein expression was increased or inhibited in response to acupuncture in NEA group. And it was decreased obviously in the hippocampus, PVN, and pituitary gland and increased obviously in the adrenal cortex in M group. Compared with the results in N group, GR protein expressions were either increased or decreased in the hippocampus, PVN, pituitary gland, and adrenal cortex by 95.99%, −23.61%, −5.2%, and 80.76% separately in NEA group. And the results were reduced significantly by 75.85%, 62.23%, and 28.04%, respectively, in the hippocampus, PVN, and pituitary gland, and the result was increased significantly by 265.01% in adrenal cortex in M group. Acupuncture induced the dual-directional regulation of GR protein expression in the hippocampus, PVN, pituitary gland, and adrenal cortex. Compared with M group, in MNEA and MEA groups, GR protein expressions were increased by 22.61% and 109.57%, 180.06% and 362.6%, and 52.9% and 246.38% separately in the hippocampus, PVN, and pituitary gland, but they were decreased by 34.82% and 88.34% in the adrenal cortex in MNEA and MEA groups. These data suggested that acupuncture promoted GR protein expression in the hippocampus, PVN, and pituitary gland but inhibited the expression in the adrenal cortex. The results in MEA were superior to MNEA group.

### 3.3. Effect of Acupuncture on CRH Protein Expression Induced by UCMS Stimulation (Figure 4)

Western blot analysis showed that there was a detectable signal of CRH in the hippocampus, PVN, and pituitary gland. CRH protein expression was slightly increased in response to acupuncture in NEA group. And the remarkable increase of CRH protein expression was discovered in M group. Obviously, acupuncture downregulated CRH protein overexpression induced by UCMS stimulation. Compared with N group, CRH protein expressions were increased or inhibited in NEA group in the hippocampus, PVN, and pituitary gland by 109.03%, 67.31%, and −23.15% separately. And the results were increased significantly in the hippocampus, PVN, and pituitary gland in M group by 543.81%, 307.66%, and 89.18%. The UCMS-induced overexpression of CRH protein could be inhibited by acupuncture. Compared with M group, CRH protein expression was reduced remarkably in the hippocampus, PVN, and pituitary gland in MNEA and MEA groups by 36.91% and 86.95%, 20.86% and 53.67%, 24.64% and 65.03% successively. Those data indicated that acupuncture inhibited CRH protein expression in the hippocampus, PVN, and pituitary gland. The results in MEA group were superior to MNEA group.

### 3.4. Effect of Acupuncture on ACTHR Protein Expression Induced by UCMS Stimulation (Figure 5)

ACTHR is ACTH receptor. Western blot analysis showed that there was a detectable signal of ACTHR in the pituitary gland and adrenal cortex. ACTHR protein expression was slightly inhibited in response to acupuncture in NEA group. The remarkable increase of ACTHR protein expression was discovered in M group, and acupuncture downregulated obviously ACTHR protein overexpression induced by UCMS stimulation. Compared with N group, ACTHR protein expression was slightly inhibited in NEA group in the pituitary gland and adrenal cortex by 10.6% and 29.61% separately; ACTHR expression was increased significantly in the pituitary gland and adrenal cortex in M group by 95.78% and 123.79%. Compared with M group, ACTHR protein expression was reduced remarkably in pituitary gland and adrenal cortex in MNEA and MEA groups by 32.9% and 69.38% and 51.52% and 71.58% successively. Those data indicated that acupuncture inhibited ACTHR expression in the pituitary gland and adrenal cortex. The results in MEA group were superior to MNEA group.

## 4. Discussion

The main results of the study were as follows. (1) The UCMS model rats showed the decrease of the activity and sucrose consumption, explaining the success of stress model in the study. (2) UCMS induced the increase of GR protein expression in adrenal cortex and the decrease in the hippocampus, PVN, and pituitary gland. Acupuncture induced the dual-directional regulation on GR protein expression in the hippocampus, PVN, pituitary gland, and adrenal cortex. And the effects were different in acupuncture at the different acupoints. (3) UCMS induced the increase of CRH protein expression in the hippocampus, PVN, and pituitary gland and the increase of ACTHR expression in the pituitary gland and adrenal cortex. All of those could be inhibited by acupuncture and the effects were different at different acupoints. (4) GR protein expression had been detected in the hippocampus, PVN, pituitary gland, and adrenal cortex; CRH expression had been detected in the hippocampus, PVN, and pituitary gland; and ACTHR protein expression had been detected in the pituitary gland and adrenal cortex in N and NEA groups. But the above corresponding expressions could be either increased or decreased in NEA group as compared with N group. Except that there were significant differences in GR and CRH protein expressions in hippocampus and CRH protein expression in the PVN between the two groups, the differences were not significant in the rest aspects.

### 4.1. Behavior Changes of UCMS Model Rats

After modeling, the horizontal, vertical, and along-the-line movements of the UCMS model rats were decreased apparently as compared with the normal model rats and the sucrose consumption was reduced significantly. These results were similar to the behavior changes of the chronic stress model rats in the previous studies [22]. It was explained that the stress model set up in this study was successful.

### 4.2. The Dual-Directional Regulation of Acupuncture on GR Protein Expression Induced by UCMS Stimulation

The chronic stress induced the decrease of GR expression in many organs of the body and the increase of adrenal weight. Acupuncture relieved the overexcitation of HPAA function induced by chronic stress [23, 24]. But the changes in GR protein expression caused by acupuncture were not clear completely in terms of physiology and pathology. The hazard caused by stress reaction is due to the higher concentration of GC in the body. The affinity of GR was very low with GR, and GR was only activated under the high concentration of GC [25]. GR participates in mainly the negative feedback regulation on HPAA [26].

The adrenal cortex is the target organ of the synthesis and secretion of GC. The long-term chronic stress stimulation can cause the excessive secretion of GC in the adrenal cortex of the rat. This study observed that UCMS induced the significant decrease of GR protein expression in the hippocampus, PVN, and pituitary gland. Acupuncture upregulated GR protein expression in the hippocampus, PVN, and pituitary gland. Perhaps, through promoting the connection of high concentration GC with GR in the hippocampus, PVN, and pituitary gland, the function of GR was activated and the negative feedback regulation of GR was intensified on HPAA. The decrease of GC level caused the decrease of HPAA excitability. The decrease of GC level lowered the combination with GR in adrenal cortex; as a result, GR activity as well as its protein expression was decreased. The results above were caused by the inhibition of acupuncture on GR protein expression induced by UCMS stimulation in the adrenal cortex. It was indicated that acupuncture presented the dual-directional regulation on GR protein expression induced by UCMS stimulation, which was achieved via HPAA. Our preliminary work had observed the impacts of acupuncture on the peripheral blood CORT in the rats with the spinal cord injury [15] and it was discovered that acupuncture promoted the increase of CORT level via the spinal reflex. Acupuncture inhibited the increase of GR level in UCMS model rats via the regulation of HPAA function. The acupoint specificity effect had been verified molecularly via the different expressions of GR protein in the hippocampus, PVN, and pituitary gland induced by acupuncture at different acupoints.

### 4.3. Inhibition of Acupuncture on CRH Protein Expressions Induced by UCMS Stimulation

Being the most superior hormone in HPAA, CRH launches the response of HPAA to the corresponding stressor. Simultaneously, CRH is also involved in the activation in the sympathetic-adrenal-medullary system. Therefore, it is recognized generally that CRH is one of the important initiating factors in the stress process. The increase of CRH secretion may be used as an objective index to reflect the body's stress state [27, 28]. This study observed that acupuncture decreased UCMS-induced overexpression of CRH protein. Possibly, acupuncture promoted GR protein expression of CRH neurons in the hippocampus, PVN, and pituitary gland and downregulated the excitability of CRH neurons so as to reduce the protein expression of CRH. The acupoints specificity effect of the acupuncture regulation on HPAA function via the difference protein expressions of CRH in the hippocampus, PVN, and pituitary gland by acupuncture at different acupoints was further verified.

### 4.4. Inhibition of Acupuncture on the ACTHR Protein Expression Induced by UCMS Stimulation

Lian et al. had made the model fitting to explain the stress reaction impact on the interaction of GR and ACTHR gene with stress factors. The results indicated that GR and ACTHR gene variants were the contributing factors of the decline of psychological stress reaction, physiological stress reaction, and work ability. It showed that ACTHR expression was closely related to the stress reaction [29].

This study had detected the protein expression of ACTHR in the pituitary gland and adrenal cortex and observed that UCMS induced the increase of ACTHR protein expression in the pituitary gland and adrenal cortex, which was downregulated by acupuncture. Additionally, the effects were different on the different acupoints, which was likely related to the downregulation of acupuncture on CRH protein expression and the inhibition of ACTHR protein expression via HPAA.

This study observed that acupuncture impacts the protein expressions of CRH, ACTHR, and GR in the normal model rats. Except for the significant differences in the protein expressions of GR and CRH in the hippocampus and the protein expression of CRH in the PVN between the N group and NEA group, the differences in the rest aspects were not significant. The stress is the comprehensive reaction of the body to the stressor [30]. Under the stimulation by stressor, the body generates a series of neural endocrinal reactions via HPAA excitation so as to enable the body to enhance the resistance and maintain and recover the internal stability under the specific situation [31]. Being a kind of stress stimulation, acupuncture activated the activity of brain central CRH neurons of the normal model rats. But since HPAA function was of the normal state in the normal animal, the stress state can be relieved rapidly. Therefore, this stress state was just manifested in the relevant molecules of the brain center. Additionally, the increase of GR protein expression in the hippocampus could promote the growth and development of the hippocampal neuron [32]. It was explained that acupuncture presented the protective effect on the central nerve in the normal model.

## 5. Conclusion

Acupuncture improved the behavior changes induced by UCMS stimulation, which was related to the promotion of acupuncture on the combination of GC and GR in the hippocampus, PVN, and pituitary gland, the activation of GR, and promotion of GR protein expression. The increase of GR protein expression induced the negative feedback inhibition on CRH protein expression, downregulated ACTHR overexpression in the pituitary gland and adrenal cortex, decreased GC level, and reduced GR activity in the adrenal cortex and the protein expression. Molecularly, it had been verified that the regulation of acupuncture on stress reaction was achieved via the regulation of HPAA function, and the effects were different at different acupoints.

## Figures and Tables

**Figure 2:**
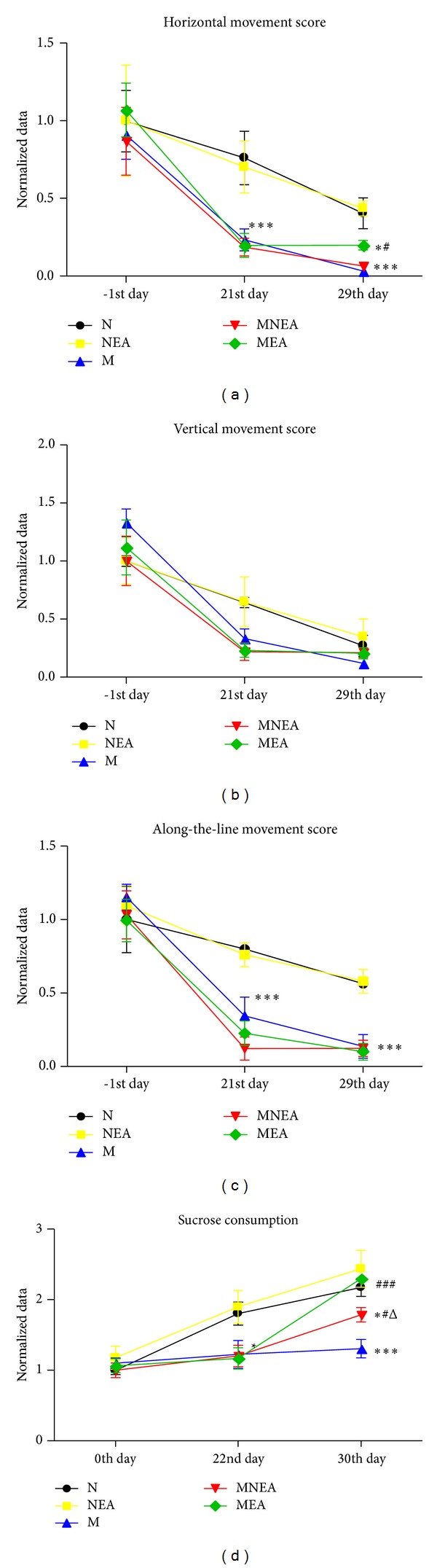
The open field test score and sucrose consumption. (a, b, and c) showed the normalized data of the horizontal, vertical, and along-the-line movement test on the −1st day, 21st day, and 29th day. (d) showed the normalized data of sucrose consumption detected on the 0th day, 22nd day, and 30th day. Normalized data [21] = tested value on the 21st, 22nd, 29th, or 30th day/tested value on the −1st day or 0th day in same animal separately. *P < 0.05, **P < 0.01, ***P < 0.001 versus N group. ^#^ *P* < 0.05, ^##^ *P* < 0.01, ^###^ *P* < 0.001 versus M Group. ^Δ^ *P* < 0.05, MNEA versus MEA (*n* = 10–12).

**Figure 3:**
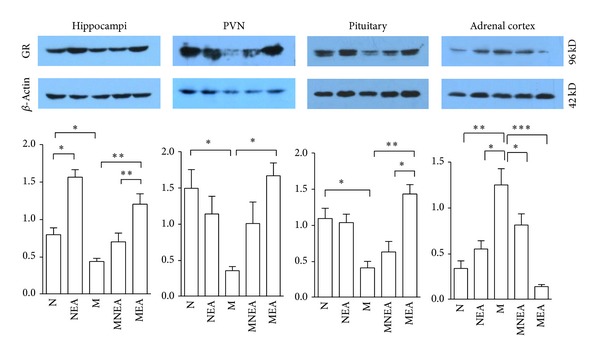
Effect of acupuncture on GR protein expression. GR protein expression was reduced obviously in the hippocampus, PVN, and pituitary gland and was dramatically increased in the adrenal cortex in M group as compared with N group. Compared with M group, GR protein expression was upregulated in the hippocampus, PVN, and pituitary gland and was downregulated in the adrenal cortex to different extents in MEAN and MNEA groups, but the results in MEA group were superior to MNEA group. The corresponding protein expression was increased obviously in the hippocampus in NEA group, which was significantly different as compared with N group. *P < 0.05, **P < 0.01, and ***P < 0.001 (*n* = 10–12) (GR: #H-300, Santa Cruz, 96KD, dilution: 1 : 1000).

**Figure 4:**
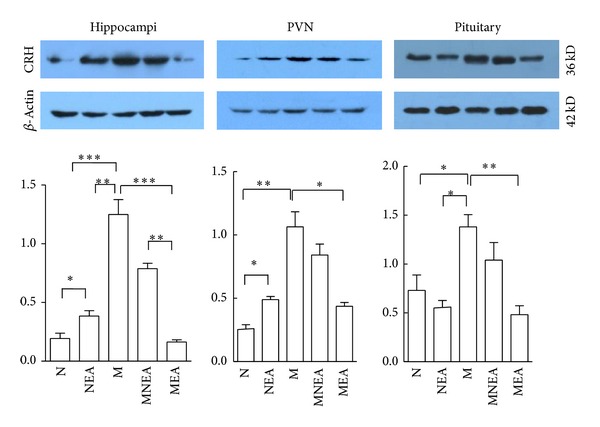
Effect of acupuncture on CRH protein expression. In M group, CRH protein expression was increased obviously in the hippocampus, PVN, and pituitary gland, which was significantly different as compared with N group. In MEA and MNEA groups, CRH protein expression was downregulated in hippocampus, PVN, and pituitary gland to different extents as compared with M group, but the results in MEA group were superior to MNEA group. The protein expression was increased significantly in the hippocampus and PVN in NEA group, which was different significantly as compared with N group. *P < 0.05, **P < 0.01, ***P < 0.001 (*n* = 10–12) (#10944-1-AP, Datashect, 36KD, dilution: 1 : 500).

**Figure 5:**
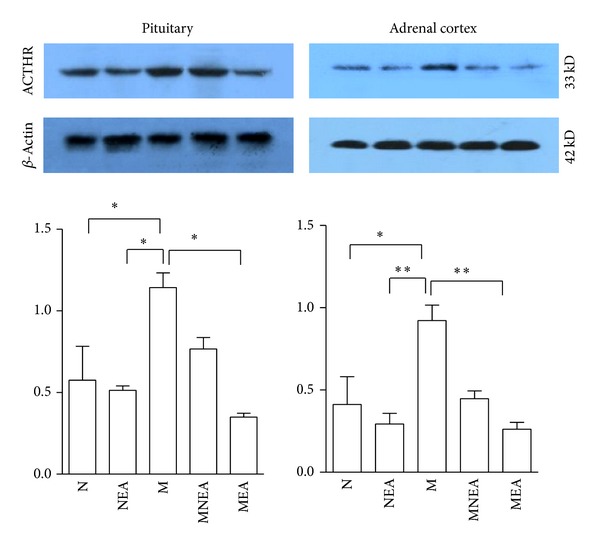
Effect of acupuncture on ACTHR protein expression. ACTHR protein expression was increased obviously in the pituitary gland and adrenal cortex in M group as compared with N group. ACTHR protein expression was downregulated in the pituitary gland and adrenal cortex in MEA and MNEA groups to different extents as compared with M group, but the results in MEA group were superior to MNEA group. The difference was not significant in comparison between NEA and N groups. *P < 0.05, **P < 0.01, ***P < 0.001 (*n* = 10–12) (#H-70, Santa Cruz, 33KD, dilution: 1 : 1000).

## Abbreviations

EA: Electroacupuncture
PVN: Hypothalamic paraventricular nucleus
HPAA: Hypothalamic-pituitary-adrenal cortex axis
UCMS: Unpredictable chronic mild stress
GR: Glucocorticoid receptor
CRH: Corticotrophin releasing hormone
ACTHR: Adrenocorticotropic hormone receptor.
